# Plasma-Enhanced Chemical Vapor Deposition of Acetylene on Codeposited Bimetal Catalysts Increasing Graphene Sheet Continuity Under Low-Temperature Growth Conditions

**DOI:** 10.1186/s11671-019-3156-y

**Published:** 2019-10-28

**Authors:** Joshua Tracy, Otto Zietz, Samuel Olson, Jun Jiao

**Affiliations:** Department of Mechanical and Materials Engineering, Portland State University, 1930 SW 4th Ave., Suite 400, Portland, OR 97201 USA

**Keywords:** Codeposition, Ni-Au bimetal catalyst, Inductively coupled plasma chemical vapor deposition (ICPCVD), Low-temperature graphene growth

## Abstract

Here, we report a novel method for low-temperature synthesis of monolayer graphene at 450 °C on a polycrystalline bimetal Ni-Au catalyst. In this study, low-temperature chemical vapor deposition synthesis of graphene was performed at 450 °C on codeposited Ni-Au which shows successful monolayer graphene formation without an extra annealing process. The experimental results suggest that electron beam codeposition of bimetal catalyst is the key procedure that enables the elimination of the pre-growth high-temperature annealing of the catalyst prior to graphene synthesis, an indispensable process, used in previous reports. The formation was further improved by plasma-assisted growth in which the inductively coupled plasma ionizes the carbon precursors that interact with codeposited Ni-Au catalyst of 50 nm in thickness at 450 °C. These combined growth conditions drastically increase the graphene’s sheet uniformity and area connectivity from 11.6% to 99%. These fabrication parameters enable the graphene formation that shifts from a bulk diffusion-based growth model towards a surface based reaction. The technique reported here opens the opportunity for the low-temperature growth of graphene for potential use in future CMOS applications.

## Introduction

It has been more than 10 years since the isolation of graphene [1], a single layer of carbon atoms in a hexagonal lattice; however, this unique 2D material has yet to be incorporated industrially to a level at which it is benefiting consumer goods. Graphene is an especially promising material for the semiconductor industry due to its notable electronic properties [2, 3]. As an atomically thin diffusion barrier [4], graphene is a powerful asset in the race to create increasingly small transistor spacings and continuing the reign of Moore’s Law. However, its applications in the semiconductor industry have been seriously hindered by the high temperatures usually required to synthesize graphene—in the range of 800 °C~ 1000 °C [5]—and the fact that the graphene transfer process is limited to planar geometries. Directly growing graphene in integrated circuits would bypass the process of transferring the graphene, another destructive process, from a growth catalyst to the device. Currently, it is possible to grow onto Cu and Ni catalysts (common metals in integrated circuits), but these require high growth temperatures [5], which could damage the already existing structures of an integrated circuit. Much research has been focused on lowering graphene synthesis temperatures and there has been a recent success. Weatherup et al. [3] have shown it is possible to grow graphene at 450 °C by adding a thermally evaporated 5 nm layer of Au on top of a sputter-deposited polycrystalline 550-nm-thick Ni catalyst, but a 600 °C pre-anneal of the Au and Ni layers is required to produce a Ni-Au alloy. A proposed mechanism is that the addition of Au to the Ni catalyst aids in limiting carbon absorption during exposure to the carbon precursor and reduces graphene nucleation and out-diffusion sites such as step edges and grain boundaries [3]. While progress has been made to reduce the synthesis temperature to the 400–600 °C range [3, 6, 7], the damaging impacts of graphene sheet transfer must also be surmounted. Direct growth onto Si or SiO_2_ is another desired target for graphene sheets, but this has not been reported in the 400 °C ~ 500 °C region needed for back-end-of-line (BEOL) semiconductor processes. While direct growth of graphene on Si or SiO_2_ has not yet been achieved, graphene synthesis on thinner catalysts represents a substantial step towards this goal. On a thinner catalyst, the resultant graphene layer is closer and closer to the target substrate. This creates the potential for developing a transfer process that is less harmful to the graphene by minimizing the amount of manual handling of the graphene due to its close proximity to the target substrate. While graphene is commonly synthesized using chemical vapor deposition (CVD) techniques, the addition of a remote plasma can help to reduce synthesis temperatures. Plasma growth energizes the precursor gases via ionization, overcoming the thermal energy that is lost when growing in the 400 °C ~ 500 °C range compared to the 800 °C ~ 1000 °C range. The advantages of a remote inductively coupled plasma are twofold: plasma is created away from the growth catalyst which reduces damage from ion bombardment on the synthesis surface, and that the plasma is produced via induction coils which are outside of the graphene growth chamber where the catalyst substrate is located. In a typical capacitive plasma system, the synthesis stage is between two metal plates inside the vacuum chamber with an electric potential between them which exposes the growth surface to any foreign material from the plasma source. Using exterior induction coil wrapped around the gas flow tube, we ionize the precursor gases by creating an alternating electromagnetic field inside the tube. This completely removes the plasma source from the chamber which does not allow for foreign material from the plasma source to potentially contaminate the growth catalyst. Here we report the synthesis of monolayer graphene (MLG) at 450 °C using inductively coupled plasma chemical vapor deposition (ICPCVD) on thin (50 nm) Ni-Au catalyst codeposited by the electron beam evaporation technique.

## Experimental Methods

### Catalyst Preparation

**Fig. 1:**
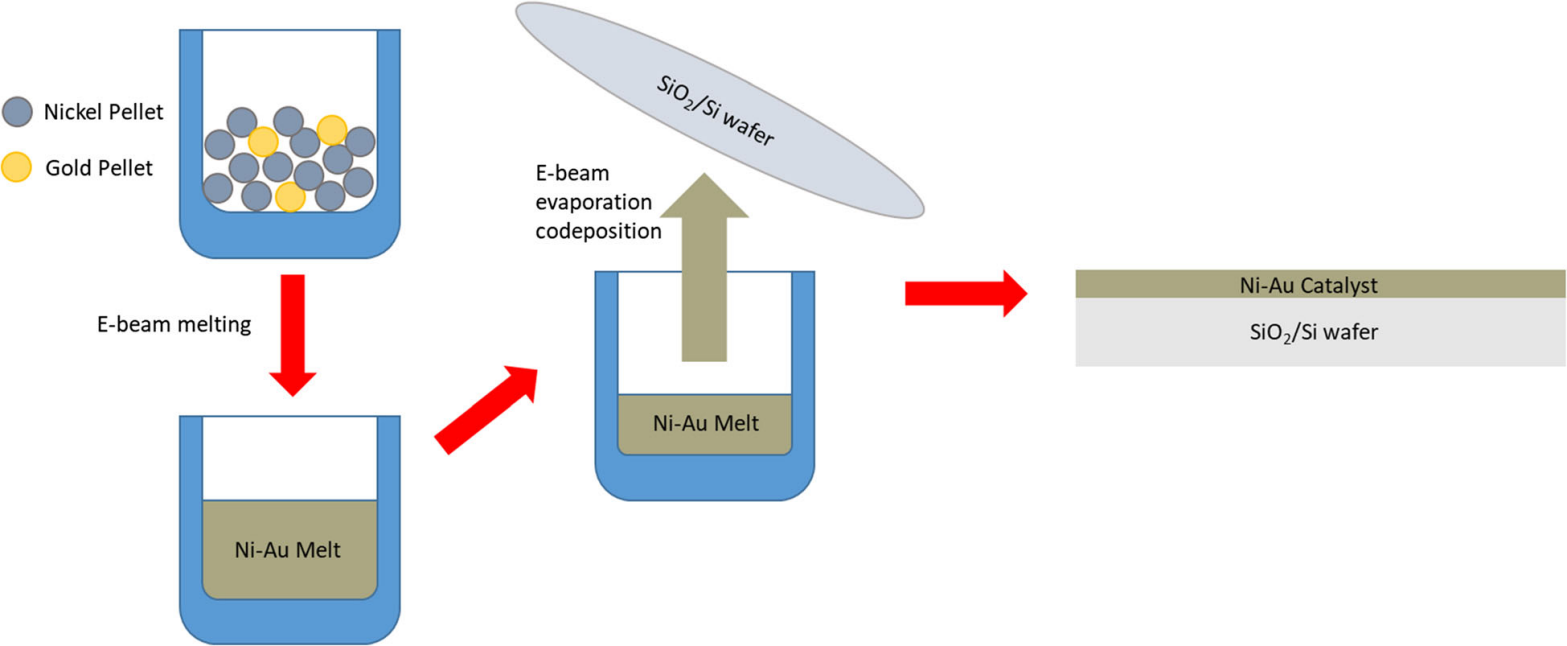
Schematic diagram of electron beam codeposition technique in which we see the Ni and Au pellets are remotely mixed in a crucible before being evaporated onto the substrate. This process serves to completely remove the necessity for pre-growth annealing used in previous literature and allows for a complete monolayer graphene synthesis at 450 °C

Two methods of catalyst preparation were used for this study to compare the effects of Ni-Au versus pure Ni, and all catalyst depositions were performed in a separate Kurt J. Lesker Physical Vapor Deposition (PVD) tool. For pure Ni graphene growth experiments, the Ni catalyst was prepared via magnetron sputtering onto SiO_2_/Si wafers to the desired thickness (50 nm). For Ni-Au catalyst preparation, Au and Ni pellets were first mixed by electron beam heating, where the electron beam system directs a beam of electrons to a crucible containing metal pellets of both Au and Ni. Electron beam deposition was preferred here due to the fact that it allows for accurate control of the weight percentage of Au in the mixture. The electron beam heats and mixes the pellets, and the resulting mixture is then codeposited via evaporation onto SiO_2_/Si wafers as schematically illustrated in Fig. 1. This remote alloying process produces a catalyst that exposes Ni to the hydrocarbon precursor while implanting Au in the Ni bulk and on the catalyst surface. The catalyst preparation which was used in the reported literature [3] deposits metals using a layering method in which multiple metals are deposited on top of each other. The layers must then be annealed at 600 °C in order to form an alloy or mixture. The codeposition technique applied in this study aimed at eliminating the catalyst annealing process and achieving graphene formation at a low temperature by using a Ni-Au alloy catalyst that was premade before the deposition. The nature of the codeposited catalyst’s pre-mixed state prevents the need for a mixing pre-anneal to form a valid catalyst. For simplicity purposes in this report, we call this catalyst deposition process “codeposition” because the Ni and Au are codeposited on to the substrate. For our experiments, we chose to deposit a 1 wt% Au alloy which has been shown to be most effective in low-temperature graphene synthesis [3]. Both pure Ni and Ni-Au catalyst were deposited to a thickness of 50 nm due to our interest in thinner catalyst growth. We found this thickness to be within previously reported ranges for nickel-based growth [8, 9], though these were at temperatures much higher than 450 °C, yet our 50 nm Au-Ni catalyst is an order of magnitude thinner than the reported 450 °C multilayer graphene (MLG) growth [3].

### Graphene Growth

Our graphene growth regimes were performed in a custom-built cold-wall ICPCVD chamber. Temperatures were measured via a thermocouple gauge affixed to the sample surface in order to detect accurate in situ surface temperatures. Gases were flown into the chamber via a showerhead. The samples were placed on a radiantly heated stage roughly 25 cm below the gas showerhead. The prepared catalysts were entered into the ICPCVD chamber and pumped to a base pressure of 1 × 10^−6^ Torr after which H_2_ was introduced at 15 sccm as the sample was heated to the process temperature of 450 °C. Once the samples reached the process temperature, the H_2_ flow was ceased, and the chamber was purged using Ar. After base pressure was reestablished, Ar was ceased and C_2_H_2_ was flown at 0.1 sccm bringing the chamber pressure to 6 × 10^−6^ Torr. For plasma-enhanced growth, RF power varying from 0 to 10 W was introduced for different growth samples. Ten watts were the highest RF power that could be supplied while maintaining a stable, remote inductively coupled plasma (ICP) since at such a low C_2_H_2_ pressure the gas resistance is very high and the induced current begins flowing elsewhere at higher plasma powers. Once the desired growth duration was reached (7 min for CVD growths and 30 s for ICPCVD growths), C_2_H_2_ flow was stopped, followed by another Ar purge to flush the remaining process gases out of the chamber. The sample heater was then shut off allowing the sample to cool to room temperature.

### Graphene Characterization

Following synthesis, graphene samples were spin-coated with PMMA. The wafers were then placed in FeCl_3_ to etch the Ni, followed by iodine/potassium iodide (40 mL H_2_O/4 g KI/1 g I_2_) solution to etch the remaining Au. The resulting film was then transferred to clean SiO_2_/Si wafers, and the PMMA was etched away with acetone. Graphene spectroscopic analysis was performed using a Horiba Jobin Yvon HR800 UV Raman spectrometer with a 532 nm laser to identify the key spectral peaks expected for graphene thin films. The D:G and D:D’ peak intensity ratios (*I*_D:G_, *I*_D:D’_) provide information about the defect density and defect type in the graphene respectively. The 2D:G peak intensity (*I*_2D:G_) as well as the 2D peak full width at half maximum (FWHM) provide information on the number of total graphene layers. Defect-free, monolayer graphene displays *I*_D:G_ of approximately 0, however, when defects are present an *I*_2D:G_ > 1.0 and FWHM_2D_ < 100 cm^−1^ are indicative of monolayer graphene [10]. Surface imaging was carried out using a Zeiss Sigma VP FEG SEM configured with an In-Lens secondary electron detector, which allows for viewing of the grain sizes and morphologies of the catalyst after the graphene formation. ImageJ was used to perform calculation of sheet percentages of greater than *I*_2D:G_ = 1 which suggests the percentage of monolayer graphene produced, and Raman peaks were fitted and analyzed using a program written in R to identify peak ratios and FWHM.

## Results and Discussion

**Fig. 2:**
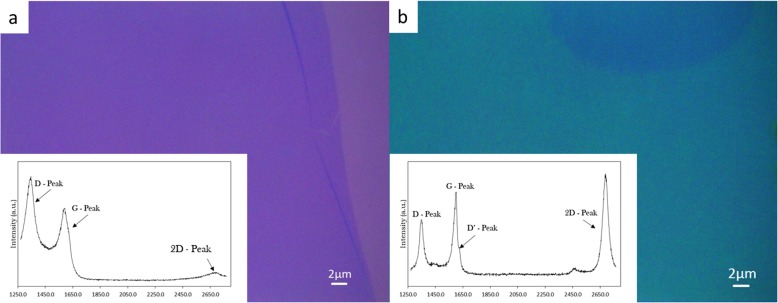
Optical images and Raman spectra (inserts) of **a** amorphous carbon as the result of an attempted graphene synthesis for 7 min C_2_H_2_ exposure on 50 nm pure Ni catalyst at 450 °C and **b** successful graphene synthesis following the same parameters as **a** on codeposited Ni-Au catalyst, please note the dark spot in the top of image **b** is an area of multilayer graphene and was captured to add contrast to help identify the surrounding monolayer area. There is an obvious improvement created by the addition of 1 wt% Au as this is the only variable changed to allow for the drastic shift from amorphous carbon to graphene. Labeled in **a** are the key peaks used for graphene characterization. For **b**, we calculate *I*_2D:G_ = 1.2, FWHM_2D_ = 48.5, *I*_D:G_ = 0.68, and *I*_D:D’_ = 5.0

**Fig. 3:**
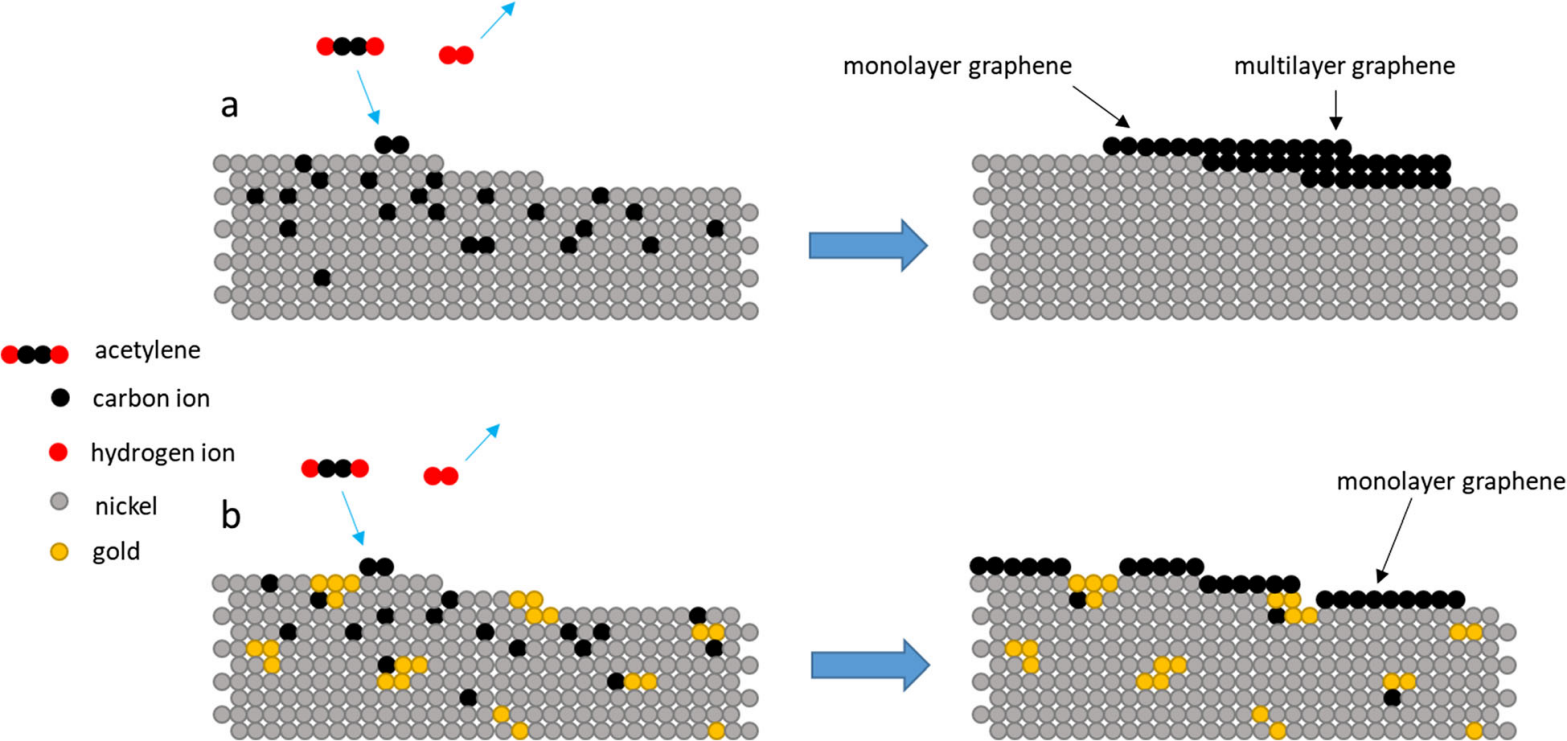
Schematic diagram of graphene growth on **a** pure Ni catalyst in which few-layer graphene (FLG) is produced due to uncontrolled C absorption and out-diffusion at high energy sites such as step edges compared to **b** codeposited Ni-Au synthesis whereby the Au acts as a C absorption limiter as well as reducing high levels of graphene production by blocking nucleation sights such as step edges

Here, it is shown that the use of codeposition of Ni-Au as a catalyst preparation technique eradicates the previously required process of 600 °C pre-annealing to alloy the Ni-Au catalyst by comparing to a baseline Ni only control catalyst and to previous reports [3]. To compare the effects of Ni-Au to pure Ni, Fig. 2 displays an average Raman spectra of transferred graphene grown via thermal CVD at 450 °C with a 7-min C_2_H_2_ exposure on (a) pure Ni and (b) the codeposited Ni-Au catalyst without annealing in contrast to previous literature [3]. The accepted CVD pure Ni catalyst growth model for graphene [11] suggests that hydrocarbon precursors are absorbed into the Ni bulk and dehydrogenated, as C shows high diffusion in Ni. During cooling, the individual C atoms diffuse out of the Ni bulk to the surface and form graphene [12]. Figure 2a displays an amorphous carbon thin film and the corresponding spectrum (insert) which is typical for low-temperature pure Ni catalyzed growth. The polycrystalline Ni catalyst contains many step edges and grain boundaries on the surface as the result of sputter deposition which act as sites that have a high probability of C diffusion and therefore as graphene nucleation sites during cooling which allows for C atoms to diffuse from the bulk in too many locations causing overlap. However, with the addition of 1 wt% Au, in Fig. 2b a drastic improvement in the Raman spectrum is observed. The spectrum shows well-defined D, G, and 2D peaks with *I*_2D:G_ = 1.2 and FWHM_2D_ = 48.5 cm^−1^ which suggests monolayer graphene formation with defects. There is a relatively large *I*_D:G_ = 0.68 and a corresponding *I*_D:D’_ = 5.0 which suggest that vacancy and lattice mismatch defect types are present [13], however, please note in both Fig. 2a, b, entire thin films were produced. Based on the literature, carbon does not readily diffuse in Au [14], and this suggests that the Au could reduce the number of graphene nucleation sites by blocking step edges and grain boundaries [3] if located in these regions producing fewer layer numbers by limiting both C absorption and out-diffusion. To illustrate the growth mechanism for this formation, Fig. 3 shows a set of schematic diagrams comparing the pure Ni vs Ni-Au growth models for thermal CVD growths, where typical Ni graphene synthesis develops multilayer graphene due to the inability to throttle the C absorption and out-diffusion (Fig. 3a), however the addition of Au aids in controlling the absorption and diffusion of C in the Ni (Fig. 3b). These results show that the addition of Au is effective in producing graphene at low temperatures which is consistent with the results reported by Weatherup et al. [3]. Most importantly, however, our results demonstrate that the use of codeposition completely eliminates the 600 °C annealing required to produce an Ni-Au alloy making this a truly 450 °C synthesis by remotely alloying the Ni and Au catalyst before deposition instead of during the growth recipe.

**Fig. 4:**
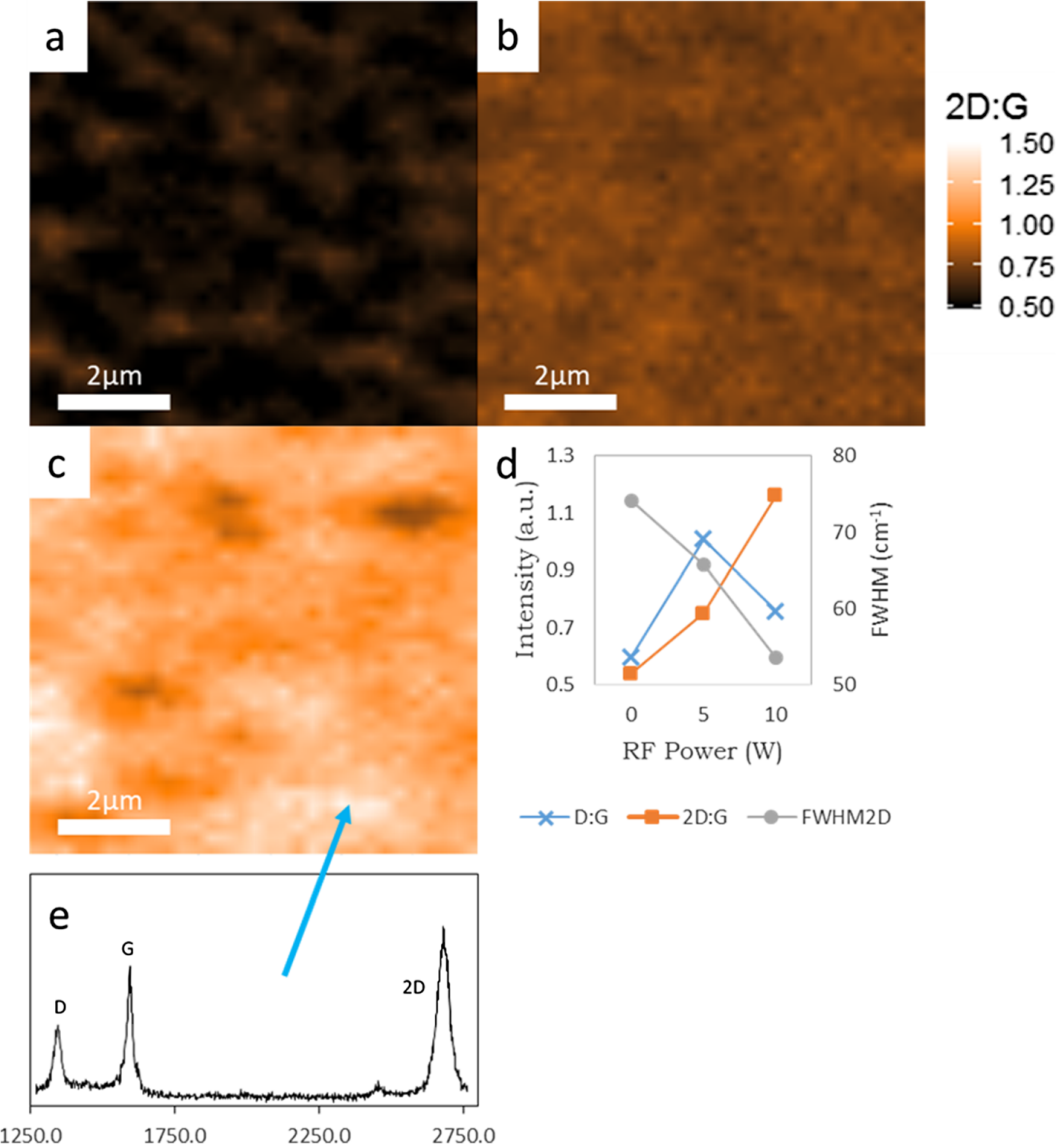
Raman maps of graphene grown on 50 nm codeposited Ni-Au with 30s C_2_H_2_ exposure time via **a** CVD, **b** ICPCVD with 5 W plasma, and **c** ICPCVD with 10 W plasma. The *I*_2D:G_, *I*_D:G_, and FWHM_2D_ for each plasma power are shown in **d** where it is apparent that 10 W serves as the best due to its higher *I*_2D:G_, lower *I*_D:G_, and smaller FWHM_2D_ compared to the others, and a representative spectra taken from the brightest region of **c** is shown in **e**

**Fig. 5:**
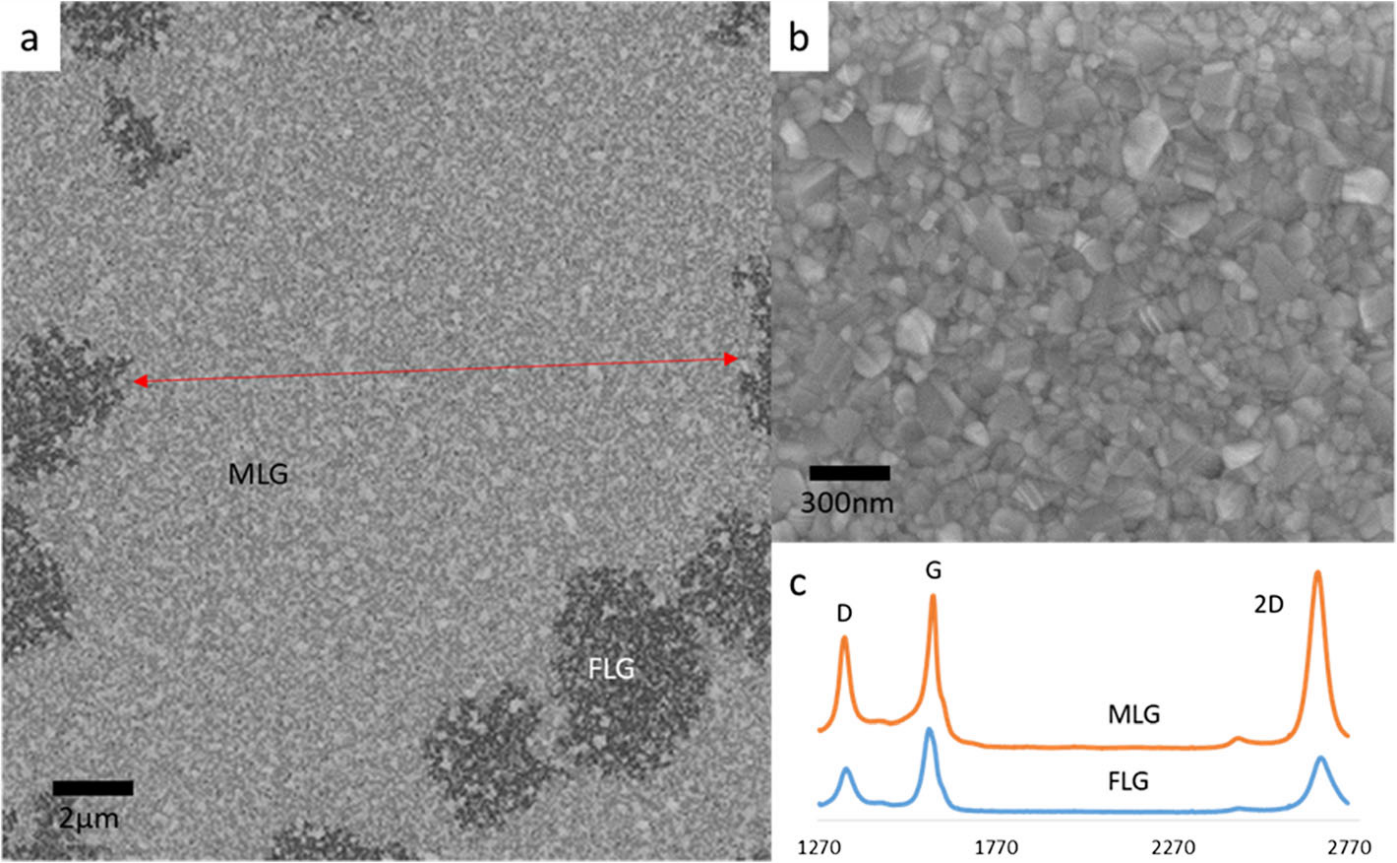
Scanning electron microscope images of as-grown graphene on codeposited Ni-Au catalyst grown at 450 °C via 10 W ICPCVD which show **a** a 15 μm wide section of continuous monolayer graphene (MLG) (red arrow) with few-layer graphene (FLG) islands (dark) (corresponding averaged Raman spectra in **c**) and **b** high magnification of monolayer graphene area where the graphene is formed on the top of the catalyst grains

**Fig. 6:**
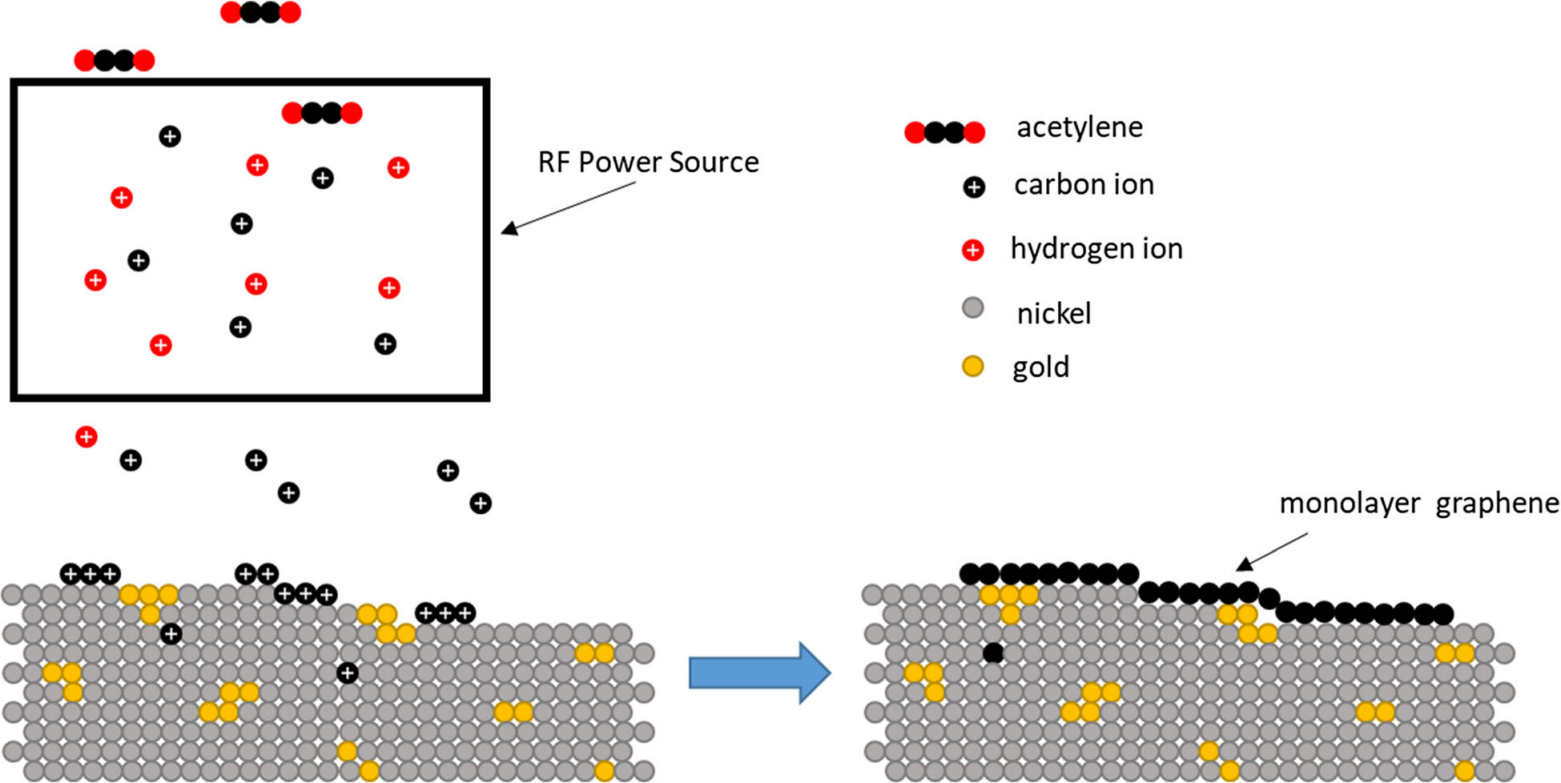
Schematic diagram of ICPCVD growth in which we see a shift towards a surface dominated mechanism. This shift is caused by ionization of C atoms that act as high-energy graphene nucleation sites once they reach the catalyst surface and allows for the use of a thinner catalyst as there is less absorption which leads to overproduction of graphene during cooling

While codeposited Ni-Au does produce graphene films, it is crucial to be able to produce large areas of continuous and uniform graphene for practical applications. To address this issue, three growths on codeposited Ni-Au were performed to compare the effects of incorporating a remote plasma with three different plasma powers into the growth. Figure 4a–c shows a Raman *I*_2D:G_ map of codeposited Ni-Au grown graphene via ICPCVD (0 W, 5 W, and 10 W respectively) with a 30s C_2_H_2_ exposure time. Figure 4a is a Raman map of synthesis performed without the addition of RF plasma power which displays a checkerboard pattern that is the result of small areas of alternating graphene layer thickness. The addition of RF plasma power of 5 W in Fig. 4b and 10 W in Fig. 4c shows larger portions of connected, more uniform layers displayed by an increase in area of uniform *I*_2D:G_, represented by large areas of uniform color, with increasing RF power. This shows that plasma addition assists in creating a larger, more uniform graphene sheet which is further supported by the data chart in Fig. 4d. The trend is as the RF plasma is increased to 10 W there is an increase in *I*_2D:G_, a decrease in *I*_D:G_, and a decrease in FWHM_2D_ which all are significant of monolayer graphene. To visually explore graphene thin film continuity, Fig. 5a shows an SEM image of pre-transferred graphene grown via 10 W ICPCVD where we see a 15 μm wide sheet of continuous monolayer graphene (MLG) with few islands of few-layer graphene (FLG). There is a visible speckling on the surface in Fig. 5a, but this is attributed to the underlying grain structure of the catalyst since this is an image of as-grown graphene and our polycrystalline catalyst has not been removed yet, which is detailed in Fig. 5b as well as correlating Raman spectra for the multilayer graphene (MLG) and few-layer graphene (FLG) in Fig. 5c. Our findings suggest, as represented by Fig. 6, that dehydrogenated C ions produced by the RF plasma reach the catalyst surface and act as a high-energy nucleation sites for growth to seed from. While there is absorption into the Ni, these C ions have a high probability of bonding with additional C ions on the surface producing dimers and larger molecules which are much less likely to absorb into the Ni catalyst. Since we see increased uniformity when applying plasma to the growth, and non-uniformity in thermal CVD growth, as displayed in Fig. 4, this suggests that during ICPCVD synthesis, the catalyst is not over absorbing to produce multilayer graphene. Instead, the growth was shifted to a surface-dominated mechanism. This move towards a surface-dominated growth also supports our approach of utilizing a thin catalyst which would saturate even quicker than thicker catalysts.

**Fig. 7:**
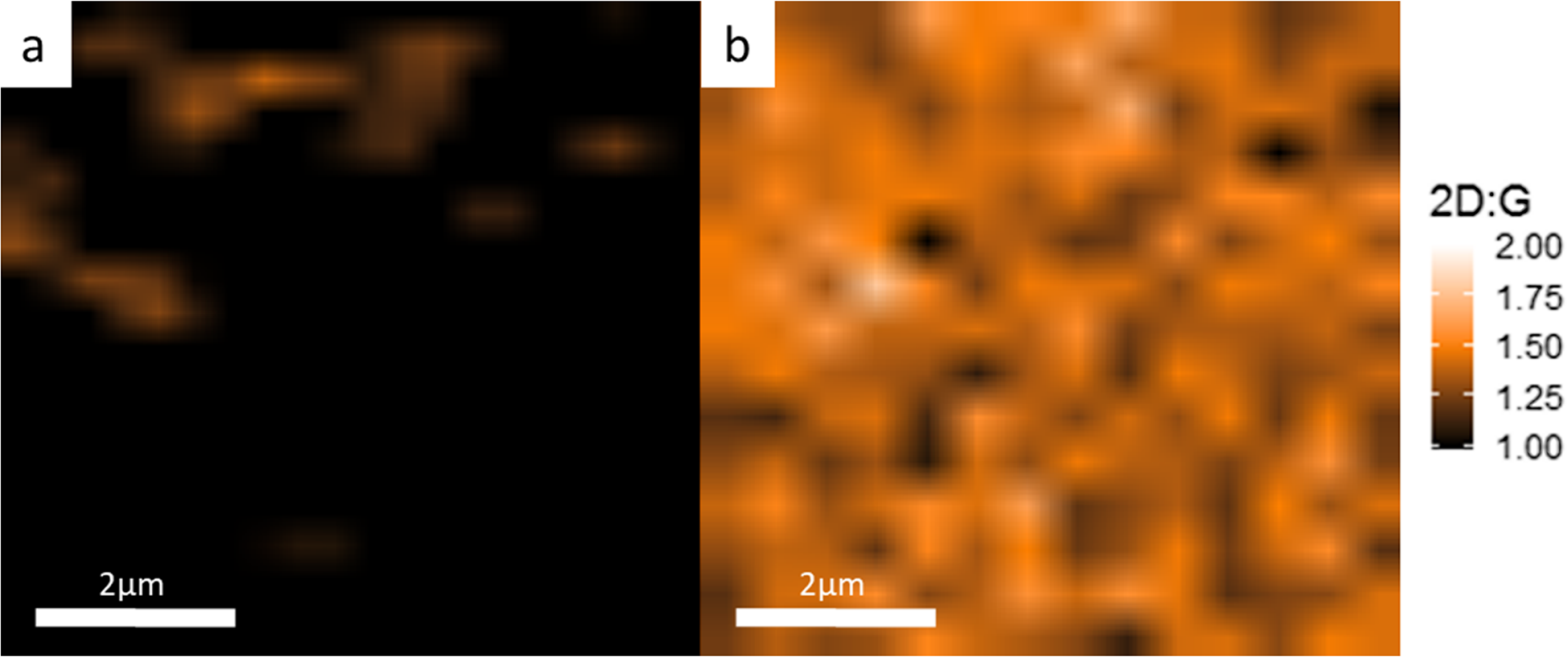
Raman maps showing *I*_2D:G_ > 1.0 for **a** CVD synthesis with 7 min exposure time versus **b** 10 W ICPCVD synthesis with 30s exposure time. Both growths are 450 °C on codeposited Ni-Au catalyst. The addition of plasma increases layer uniformity from 11.6% (**a**) to 99% (**b**)

The benefits of ICPCVD over CVD can also be seen when comparing multilayer (MLG) coverage. A set of samples from both ICPCVD and CVD syntheses was compared and the results are shown in Fig. 7 which displays Raman maps of (a) 7-min CVD growth versus (b) 30s ICPCVD growth with 10 W RF power on codeposited Ni-Au. Based on our calculation of the Raman map, we have estimated that 7-min CVD growth displays an 11.6% coverage of *I*_2D:G_ > 1.0 while the 30s ICPCVD growth on the other hand displays a 99% coverage of *I*_2D:G_ > 1.0. This suggests the plasma plays an important role in graphene connectivity and uniformity on a thin catalyst and prevents the absorption of large amounts of C into the catalyst as opposed to the CVD growth in which over absorption of C leads to overproduction of graphene and thus less uniformity.

## Conclusions

We have demonstrated the validity of codeposition as a unique catalyst preparation technique which effectively removes the necessity of annealing for Ni-Au catalysts by remotely pre-alloying the catalyst during e-beam evaporation and producing an immediately capable catalyst for 450 °C graphene growth. The addition of using an inductively coupled plasma during the growth serves to increase graphene thin film area and layer uniformity by shifting the synthesis process to a surface dominated mechanism which is beneficial when thin catalysts are used for growth. The study presented here demonstrated the significant progress of using plasma-enhanced CVD and codeposited Ni-Au thin catalyst to grow graphene with improved quality at low temperature. However, the growth parameters need to be tailored and optimized with respect to the specific applications. For example, the catalyst design and optimization to further increase the graphene grain size under the low-temperature growth conditions and the direct growth of graphene on desired substrates. These are the issues that will be addressed in the ongoing investigations.

## Data Availability

All data is available from the authors via a reasonable request.

## Abbreviations

2D: Two dimensional
BEOL: Back end of line
CVD: Chemical vapor deposition
FLG: Few-layer graphene
FWHM: Full-width half maximum
ICPCVD: Inductively coupled plasma chemical vapor deposition
MLG: Monolayer graphene
PVD: Physical vapor deposition
